# Correlation between voice and life quality and occupation

**DOI:** 10.1016/S1808-8694(15)30790-4

**Published:** 2015-10-19

**Authors:** Ana Lúcia Spina, Rebecca Maunsell, Karine Sandalo, Reinaldo Gusmão, Agrício Crespo

**Affiliations:** 1 MSc; PhD student. Speech and Hearing therapist, in charge of the vocal rehabilitation program at the ENT department - HC UNICAMP; 2 Otorhinolaryngologist - Graduate Student - Assistant physician - HES-UNICAMP; 3 Speech and hearing therapist. Intern of the vocal rehabilitation program - Otorhinolaryngology Department - HC UNICAMP; 4 PhD. Professor in charge of Laryngology of the Otolaryngology Program - HC UNICAMP; 5 PhD. Professor - Head of the ENT program - HC UNICAMP

**Keywords:** dysphonia, professional, quality of life, voice

## Abstract

Dysphonia may impair the quality of communication and social relations of people, thereby directly affecting quality of life. It is common knowledge amongst professionals involved with the treatment of voice disorders the need for objective protocols to evaluate voice quality and measure its impact on the individual's quality of life.

**Aim:**

To associate life quality with the degree of dysphonia and professional voice use in a group of dysphonic patients.

**Materials and Methods:**

A prospective clinical study was undertaken with a group of dysphonic patients using an internationally validated voice-related quality-of-life protocol. A statistical analysis of the results was carried out, not distinguishing between those who use their voices professionally and those who don't.

**Results:**

dysphonia had an impact on the quality of life of all patients. There was no statistically significant difference between the groups - professional users and non-users of their voices; however, considering the groups separately, this correlation was significant only in the group of patients who do not use their voices professionally.

**Conclusion:**

Dysphonia affected the quality of life of all subjects regardless of their voice use.

## INTRODUCTION

Dysphonia, or an alteration in vocal emission is a very common disorder today^1^. It may compromise the quality of communication and, consequently, the social relationship of the individual and thus directly impact his/her quality of life. Quality of life is a concept of difficult definition, because it is considered to be subjective. After a consensus among specialists, the World Health Organization (WHO) defined quality of life as the individual's perception regarding his/her quality of life, within the cultural context and systems of values, and the relationship with the expectations, objectives and concerns. Thus quality of life is considered as the joining of multidimensional factors based on physical, mental and social parameters^2^.

Berlin and Fleck^3^ reported that quality of life has been broadly used to determine the global impact of diseases and medical treatment, considering the patient's view point.

It is a consensus among professionals who treat voice disorders that there is a need for objective protocols dealing with voice quality evaluation, which measure its implication on the patient's quality of life.

Just as hard as conceptualizing quality of life, it is to characterize the impact some disease or disorder may have on the quality of life of the individual. As far as dysphonia is concerned, many protocols have been developed in order to measure dysphonia and its impact on quality of life. In 1997, Jacobson et al.^4^ developed the “Voice Handicap Index” protocol, translated and adapted in Brazil by Behlau et al.^5^ as a protocol of “Vocal Disadvantage Index”, which can be used to measure the effectiveness of vocal treatment.

Hogikyan and Sethuraman^6^ created and validated an instrument to specifically measure dysphonia impact on the quality of life of the individual (V-RQOL-Voice-Related Quality of Life).

The protocols used to study the impact of voice disorders on the quality of life are still under initial studies^7^, thus they must be explored using different evaluation parameters.

The protocol we used in our study is the V-RQOL - Voice-Related Quality of Life - protocol, translated and adapted to Brazilian Portuguese by Mara Behlau^5^. It is a tool that considers the individual in a global manner and one that is broken down in different domains which reflect the influence of dysphonia in the physical and emotional aspects. Thus, it is possible to detect the consequences of dysphonia on the different aspects of quality of life.

Recent studies correlate quality of life to gender, quality of life and voice to functional or organic alterations^8^, quality of life and voice to cultural impact^9^, quality of life to the perception of the patient's voice and perception of the community^10^, ^11^. Nonetheless, the data relating quality of life and voice to professional activity by means of the V-RQOL - Voice-Related Quality of Life -, translated into Brazilian Portuguese as Quality of Life and Voice (QLV), is scarce.

Our questioning is if the quality of life of people who use their voices professionally can be as impacted as that of people who do not use their voices professionally, even with the same degree of dysphonia.

Thus, the present investigation aims at correlating quality of live and voice with the level of dysphonia and professional activity.

## MATERIALS AND METHODS

Our study involved 101 patients, 46 men and 55 women, all older than 18 years. All these patients first came to the otolaryngology ward with vocal complaints. They were all assessed by a physician and were referred to the vocal rehabilitation program, in the same ward. They were then invited to participate in the study and signed the informed consent form. The research project was previously approved by the Ethics in Research Committee of the Faculdade de Ciências Médicas under protocol # 261/2005. None of the patients refused to participate in the study. We included in our study all the patients who had been complaining of vocal disorders for at least two months. The patients answered the protocol V-RQOL - Voice-Related Quality of Life - translated and adapted to Brazilian Portuguese by Mara Behlau (QLV- Quality of Life and Voice), the patient was the informant. The protocol was employed immediately after medical exam and before the patient received any information about his/her laryngeal diagnosis and any voice-care instruction.

The protocol is based on questions which start by “because of my voice, how much of it is a problem” for which the subject shall answer from 5 alternatives: it never happens; it is not a problem; it doesn't happen very often; it is rarely a problem; it happens sometimes and it is a moderate problem; it happens a lot and it is almost always a problem; it always happens and it is always a significant problem. The questions explore the vocal condition associated to the patient's life situation in three domains or scores: emotional (4 questions), physical (6 questions) and the two previous ones together, called total score. The QLV protocol results in scores that go from 0 (zero) to 100; 0 (zero) is the worst indication of quality of life and 100 the best quality of life.

37 of the patients used their voices professionally, and 64 did not use their voices professionally. Voice professionals were those whom had their voices as their main work tool and who used it for at least four hours per day, without which these patients could not perform their professional activities. Among voice professionals we had teachers, telemarketing attendants, salespeople and pastors. In the group of those who did not use their voices professionally, the groups studied were: students, sewers, house wives and administrative professionals. The mean age of the voice professionals was 42 years and that of the other professionals was 51 years.

The auditory perceptive evaluation was carried out by means of consensus among three speech and hearing therapists of our ward with broad experience in the field of vocal disorders in order to grade the dysphonia. The auditory-perceptive evaluation was carried out in a sound-treated room, using the GRBAS scale and the emission of the prolonged vowel/a/. The evaluations were carried out at the time of the first interview with the patients during the time they were answering the questions of the QLV protocol.

After perception assessment, we checked to see whether there was correlation between the level of dysphonia and the results from the QLV protocol, regardless of voice use. Following that, we checked to see whether or not there was a correlation between the different domains of the QLV protocol - total, physical and emotional scores - for voice professionals and other professionals.

In order to carry out the statistical analyses, the score results were divided in three groups: group A - when the score was between 71 and 100 (better quality of life), group B - when the score was between 36 and 70 (intermediate quality of life) and group C - when the score was between 0 and 35 (worse quality of life).

As a statistical treatment, we proposed the Mantel-Haenszel chi-square exact test. The methodology considered:

– non-distinction between: voice professionals and other professionals
– distinction by professional groups.

In order to check the association between voice professionals with the level of dysphonia and also with the different scores of the QLV, we used the Chi-Square test or Fisher Exact Test.

The level of significance used was 5%.

## RESULTS

In the auditory perception evaluation of patients, regardless of the use they make of their voices, we observed the following as a result: normal voice in eight patients (7.92%), mild dysphonia in 46 patients (45.54%), moderate dysphonia in 34 patients (33.66%) and severe dysphonia in 13 patients (12.87%).

On Table I, the Fisher Exact Test (p=0.2850) led us to notice that there was no significant difference between the level of dysphonia and professional use of voice.

**Table I:** Distribution in percentage of the level of dysphonia in voice professionals and other professionals' variables and results from the Fisher Exact Test associated to the level of dysphonia and occupation.

Level of dysphonia	Voice professionals (%)	Other professionals(%)
Absent	5.41	9.38
Mild	48.65	43.75
Moderate	40.54	29.69
Severe	5.41	17.19

Fisher Exact Test p= 0.2850

Table II shows the percentage of dysphonic subjects in the Quality of Life scores were broken down in groups A, B and C, in relation to the professional use of voice. The statistical result provided by the chi-square test shows that there is no statistical difference between voice professionals and those who do not use their voices to perform their professional activity. We also observed that the perceptual distribution of the individuals in each group A, B and C were similar for the total score, the physical and emotional one for both groups.

**Table II:** Percentage distribution of the subjects in the Quality of Life scores in relation to their professional use of voice, broken down into groups A, B and C.

	Total score		Emotional score		Physical score	
	VP	NPV	VP	NPV	VP	NPV
Group A	48.6	61	64.9	59.4	45.9	59.4
Group B	37.9	26.5	24.3	25	35.2	28.1
Group C	13.5	12.5	10.8	15.6	18.9	12.5

VP: voice professional; NPV: other professionals

Chi-square test: p-value for total score =0.4455

p-value for the emotional score =0.7744

p-value for the physical score =0.4061

Table III shows the general percentage of the degree of dysphonia of all the subjects associated to the Quality of Life Scores in Groups A, B and C. On this Table, Mantel-Haenszel exact chi-square test proved to be statistically significant for the physical score. The total score and the emotional one were close to significance, with p values of p=0.0013 and p=0.0540, respectively. These results reveal that there is a correlation between the level of dysphonia and the quality of life in the different scores investigated, regardless of the use they make of their voices.

**Table III:** General percentage of the distribution of subjects, regardless of their voice use, in the difference Quality of Life scores associated with the level of dysphonia of all the subjects.

	Group A			Group B			Group C		
	ST	SE	SF	ST	SE	SF	ST	SE	SF
Absent	10.5	11.3	10.9	6.4	4.0	6.4	0.0	0.0	0.0
Mild	50.9	48.4	54.5	48.3	44.0	48.3	15.3	35.7	6.7
Moderate	31.6	30.7	30.9	32.3	32.0	25.9	46.2	50.0	60.0
Severe	7.0	9.6	3.7	13.0	20.0	19.4	38.5	14.3	33.3

ST: total score; SE: emotional score; SF: physical score

Mantel-Haenszel chi-square: p-value for a total score =0.0013

p-value for the emotional score =0.054

p-value for physical score =<0.0001

We have associated the Quality of Life Scores to the level of dysphonia distinctly for voice professionals and other professionals (Table IV). We observed a statistical significance of the total and physical scores for other professionals. These results, revealed by the Mantel-Haenszel chi-square exact test point to an association between the level of dysphonia and the quality of life for the those who do not use their voices professionally. For the voice professional such correlation was not significant.

**Table IV:** Results from the Mantel-Haenszel chi-square test which relates the Life Quality scores to the level of dysphonia in voice professionals and non-voice professionals.

	Voice professionals	Non-voice professionals
	p-value	p-value
Total score	0.2412	0.0032
Emotional score	0.4921	0.0905
Physical score	0.0858	0.0001

## DISCUSSION

The life quality of dysphonic patients reflect their physical and emotional possibilities during dysphonia. This topic has received special health-care attention^1^. Protocol QLV has been increasingly used to associate dysphonia to the difficulties of the patient's day-to-day life. It is divulged as a protocol of easy and fast application, which makes it practical^12^. This type of evaluation is a precious component added to the contemporary procedures used to assess quality of life alterations stemming from vocal alterations, because protocols which assess general health did not show sensitivity enough to detect vocal limitations^6^.

The patients we studied were diagnosed with different vocal fold alterations, all benign.

Although those who do not use their voices professionally have remained dysphonic (63.37%), we had a large number of patients who depend professionally on their voices. None of the patients used their voices artistically. We believe that the large number of patients who made professional use of their voices in this study is associated with the need for specific care which also reflects the specificity of this field. We deemed voice professionals, all of those to whom voice is their main working tool and for whom voice use was paramount in their professional activities for at least four hours a day. In these cases, voice alteration or aphonia prevents the work activity.

As far as the degree of dysphonia is concerned, the greatest concentration happened in mild dysphonia, regardless of professional voice use. Although this concentration in mild dysphonia did not show statistical significance, our study reinforces that dysphonia has so much impact on life quality that make the individual seek help even with mild voice alterations. Severe dysphonia happened in a greater percentage to those who do not use their voices professionally, very likely because of the limitation that severe dysphonia causes and the correlation with the incapacity to use the voice in professional activities in severe dysphonic spells. A voice professional with severe dysphonia is unable to act professionally, and this makes him/her look for help before the dysphonia worsens.

In order to carry out the statistical study, we grouped the results from the total, emotional and physical scores. Group A was characterized as the group with score results from 71 to 100, Group B had results between 36 and 70 and Group C had results between 0 and 35. Results from the questionnaire scores can be used only to compare the subjects, the closer to 100, the better the quality of life. Group A was, therefore, the group with the best quality of life, Group B had intermediate quality of life and Group C had the worst impact on their quality of life. Table II shows the correlation results of scores: total, physical and emotional, with Groups A, B and C differentiating the professional groups showed that the differences among them were not significant. There was a similar distribution considering the three scores in voice professionals and other professionals. This suggests that the quality of life is impaired in a similar way both for voice professionals and other professionals who seek medical care on account of dysphonia.

On Table III we did not consider the difference among the groups of professionals with the aim of observing if, in general, the entire set of patients had any tendency. We observed that the worse the dysphonia, the worse was the quality of life. Altered voice alters the quality of life, regardless of being a voice professional or not. Nonetheless, the physical score had statistical significance when we associated the degree of dysphonia with the quality of life, in other words, the worse the dysphonia, the worse was their physical score, regardless of voice use. The total and emotional scores came close to significance, and this indicates a correlation tendency between worse voice quality and worse total and emotional scores. It is very likely that this result correlates to the fact that physical score issues are more easily observed and thus identified by the patient, since these are physical sensations, more objective, while the emotional score can vary or even be difficult to measure when the sensation is not present at the time of assessment.

Some authors suggest that a voice problem impairing the career of a voice professional may even not be perceived by another individual who does not make a professional use of his voice6. Nonetheless, our study suggests that when the voice alteration is a complaint that causes the patient to look for help, the quality of life is altered as much as the quality of life of someone who makes a professional use of his/her voice.

Table IV shows the results of the Mantel-Haenszel chi-square exact test which correlates the quality of life scores to the level of dysphonia in voice professionals and other professionals. There was statistical significance for the other professionals in the scores: total (p=0.0032) and physical (p=0.0001). This suggests again that the physical score is more easily recognized by dysphonic subjects. It is likely that this same significance would not show in voice professionals because there is not so much of a difference from the physical to the emotional scores which may be compromised. Results did not reveal significant differences among voice professionals' scores. For other professionals, the worse the level of dysphonia, the worse the total and physical scores.

Given the results from this study, we believe it is worth reflecting at the moment of treatment in regards of the people who do not use their voices professionally. Although intuitively we have the impression that the impact of dysphonia on a subject who uses his voice professionally can be more relevant than dysphonia on a person who does not use the voice professionally, this study suggests that this is not necessarily true. Especially in regards of the surgical proposals in laryngology, we believe expectations must be well clarified and discussed with the patient, having that the impact of an unsatisfactory result can mean important losses both for the voice professional as for those individuals who do not use their voices professionally.

As it happens to other fields of medicine, with the growing demand for evidence-based results, it is paramount to describe results which are reliable and comparable. Today, there are numerous research designs and tools which can be used to describe vocal results obtained from surgical and speech therapy treatments, nonetheless, very little comparable data that can be correlated. We believe that in the future, the QLV protocol should be included among these tools as a means to assess the vocal disorder impact on the lives of our patients, and specially on the report of vocal disorders'treatment.

## CONCLUSIONS

From the study of a group of dysphonic individuals studied through the V-RQOL protocol, we have concluded that dysphonia has impaired their quality of life equally, regardless of the use they make of their voices. The level of dysphonia was associated with a worse quality of life, regardless of the person's occupation. When we considered the difference between voice professionals and those who do not use their voices professionally, we noticed a significant statistical correlation between quality of life and the level of dysphonia only in the group of patients who do not use their voices professionally.
